# The “cytokine storm” in infection and sepsis: win the battle but lose the war

**DOI:** 10.1186/s40779-025-00678-0

**Published:** 2026-01-12

**Authors:** Jiang-Bo Fan, Qin-Yuan Li, Xi-Feng Feng, Si-Yuan Huang, Rui Wang, Feng-Ying Liao, Di Liu, Wen-Yi Liu, Jian-Hui Sun, Hua-Cai Zhang, Hui-Ting Zhou, Jian-Xin Jiang, Zhen Wang, Ling Zeng

**Affiliations:** 1 Department of Trauma Medical Center, Daping Hospital, State Key Laboratory of Trauma and Chemical Poisoning, Army Medical University, Chongqing, 400042 China; 2 Department of Respiratory Children’s Hospital of Chongqing Medical University, National Clinical Research Center for Children and Adolescents’ Health and Diseases, Ministry of Education Key Laboratory of Child Development and Disorders, Chongqing Key Laboratory of Child Rare Diseases in Infection and Immunity, Chongqing, 400014 China; 3 Institute of Pediatric Research, Children’s Hospital of Soochow University, Suzhou, 215025 Jiangsu China; 4 Department of Critical Care Medicine, State Key Laboratory of Trauma, Burns and Combined Injury, Daping Hospital, Army Medical University, Chongqing, 400042 China

**Keywords:** Cytokine storm, Inflammatory response, Severe infection, Sepsis, Multiple organ dysfunction syndrome

## Abstract

The cytokine storm, a life-threatening systemic inflammatory syndrome, is the primary driver of multiorgan failure in different clinical situations, including severe infections, autoimmune diseases, chimeric antigen receptor (CAR) T cell immunotherapy for cancer, and genetic syndromes. This review focuses primarily on cytokine storms triggered by severe infections such as viral pneumonia and bacterial sepsis, and explores the underlying mechanisms of cytokine storms and potential therapeutic interventions. Cytokine storms are characterized primarily by the excessive release of proinflammatory cytokines, which are triggered by pathogen-associated molecular patterns (PAMPs), damage-associated molecular patterns (DAMPs), and PANoptosis, all of which activate immune signaling cascades. Amplification mechanisms involve positive feedback loops and the failure of negative feedback mechanisms, leading to uncontrolled inflammation. Like a pyrrhic victory, the excessive activation of the immune system eliminated invading pathogens but caused catastrophic damage due to multiple organ dysfunction syndrome (MODS), turning the life-saving response into a life-threatening war. Therapeutic strategies, including cytokine antagonists, Janus kinase (JAK) inhibitors, caspase inhibitors, glucocorticoids, and blood purification therapies, aim to interrupt the self-amplifying cycle of inflammation that propagates organ injury, thereby reducing MODS and mortality. Challenges include optimizing the treatment timing and patient stratification. Future research should focus on combination therapies and personalized medicine based on the heterogeneity of infections and sepsis. Advances in multiomics and targeted therapies provide new hope for managing infections and sepsis.

**Supplementary Information:**

The online version contains supplementary material available at 10.1186/s40779-025-00678-0.

## Background

The cytokine storm, also known as cytokine release syndrome (CRS), is a life-threatening systemic inflammatory condition characterized by the excessive and uncontrolled release of proinflammatory cytokines and chemokines, leading to a hyperactive immune response that paradoxically damages host tissues and organs, even resulting in death [1–4]. Under physiological conditions, cytokines such as interleukin (IL)-6, IL-1β, tumor necrosis factor-α (TNF-α), and interferon-γ (IFN-γ) play critical roles in coordinating immune defenses against invading pathogens, promoting tissue repair, and maintaining homeostasis [5, 6]. However, during a cytokine storm, the regulatory mechanisms that balance immune activation and suppression fail, triggering an uncontrolled inflammatory response [7, 8]. This dysregulation triggers widespread inflammation and parenchymal cell damage, which leads to vascular leakage, multiple organ dysfunction, and even death. Cytokine storms cause catastrophic immune dysregulation, in which an uncontrolled immune response drives systemic dysfunction [9, 10].

Cytokine storms can occur in different clinical situations, including severe infections, autoimmune diseases, chimeric antigen receptor (CAR) T cell immunotherapy for cancer [11–13], and genetic syndromes. Infectious diseases are among the most common triggers. Viral infections such as severe acute respiratory syndrome coronavirus (SARS-CoV) are frequently associated with severe cytokine storms, particularly in critically ill patients [14]. The virus initiates this cascade via spike protein binding to angiotensin-converting enzyme 2 (ACE2) receptors, activating innate immune pathways such as Toll-like receptors (TLRs) and inflammasome signaling. This process triggers the excessive production of proinflammatory cytokines, including IL-6, IL-1β, and TNF-α [15–17], contributing to acute respiratory distress syndrome (ARDS) [18, 19], which is characterized by pulmonary edema, hypoxemia, and respiratory failure. Similarly, highly pathogenic influenza viruses, including Hemagglutinin type 1 and neuraminidase type 1 (H1N1) [20, 21], induce fatal hypercytokinemia by damaging respiratory epithelial cells and releasing damage-associated molecular patterns (DAMPs). These DAMPs activate alveolar macrophages and dendritic cells (DCs), resulting in the excessive release of cytokines that exacerbate lung injury and systemic inflammation. Other viral infections, such as Ebola and Lassa fever, can also provoke cytokine storms through direct immune cell infection or excessive IFN signaling, leading to vascular leakage, coagulopathy, and shock [22–24].

Bacterial infections, particularly sepsis caused by Gram-negative bacteria such as *Escherichia coli* or *Klebsiella pneumoniae*, constitute another major cause of cytokine storms [25, 26]. Bacterial lipopolysaccharide (LPS) binds to Toll-like receptors 4 (TLR4) on macrophages and DCs, activating the nuclear factor-kappa B (NF-κB) and mitogen-activated protein kinase (MAPK) signaling pathways [27]. This process triggers the release of IL-6, IL-1β, and TNF-α, which drive systemic inflammation, sepsis, septic shock, and disseminated intravascular coagulation (DIC). In toxic shock syndrome, bacterial superantigens from *Staphylococcus aureus* or *Streptococcus pyogenes* bypass normal antigen presentation [28, 29], nonspecifically activating large populations of T cells. These cells release large amounts of IFN-γ and IL-2, leading to hypotension, rash, and multiorgan failure. Parasitic infections, such as severe malaria caused by *Plasmodium falciparum*, can also induce cytokine storms. Hemozoin, a byproduct of parasite metabolism, activates the NOD-like receptor thermal protein domain-containing protein 3 (NLRP3) inflammasome in macrophages, promoting IL-1β secretion and disrupting the blood-brain barrier in individuals with cerebral malaria [30]. In addition, macrophage activation syndrome (MAS) [31], phagocytic lymphohistiocytosis (HLH) [32], CAR-T cell immunotherapy [33, 34], graft-versus-host disease (GVHD) after allogeneic hematopoietic stem cell transplantation [35], and acquired dysfunction of the NLRP3 gene leading to cryopyrin-associated periodic syndrome can also cause cytokine storms [36].

The adage “the road to hell is paved with good intentions” aptly describes the paradoxical nature of inflammatory responses in sepsis. Although inflammatory cytokine responses are crucial for protective immunity, pathogens such as bacteria, viruses, fungi, and parasites can provoke excessive responses known as “cytokine storms”. These storms are detrimental to the host and may lead to multiorgan failure and mortality. The cytokine storm, or CRS, is a life-threatening inflammatory condition characterized by elevated levels of circulating cytokines and the overactivation of immune cells [37, 38]. In this article, we review the underlying mechanisms and advances in therapies for cytokine storms occurring in individuals with sepsis and lethal infections, aiming to provide new insights for clinical diagnosis and treatment. This paradox, eliminating pathogens ("winning the battle") while triggering multiple organ dysfunction syndrome (MODS) ("losing the war"), defines the essence of the cytokine storm. Understanding where and how the immune system shifts from defense to self-destruction is the central challenge addressed in this review.

## Progress in defining the cytokine storm

In 1993, Ferrara et al. [39] first introduced the term "cytokine storm" during their investigation of acute GVHD. Their study revealed that IL-1 plays a central role in the cytokine storm associated with GVHD. IL-1 not only directly activates immune cells but also promotes the release of other cytokines, such as IL-6 and TNF-α, thereby further exacerbating the inflammatory response. These findings suggest that inhibiting IL-1 activity could decrease the severity of GVHD. In 1996, Aikawa et al. [40] applied this term to describe the systemic inflammatory response observed in individuals with sepsis and MODS following surgical trauma. In the early twenty-first century, the cytokine storm was considered a flu-like syndrome that developed after severe infections and immunotherapies [41]. During the outbreaks of severe acute respiratory syndrome in 2003, H5N1 influenza in 2005, and H1N1 influenza in 2009 [42], the cytokine storm was used to describe the phenomenon of the excessive production of inflammatory cytokines following infection [43, 44].

In 2010, Morgan et al. [12] reported that elevated serum cytokine levels were associated with multiorgan failure following CAR-T cell immunotherapy and termed this phenomenon the "cytokine storm". Many CAR-T cells rapidly accumulate in the lungs after infusion and are activated via the recognition of ErbB2 receptor tyrosine kinase 2 (ERBB2) and release cytokines. Patients experienced typical symptoms of the cytokine storm, such as high fever, hypotension, dyspnea, and pulmonary edema. In 2018, a systematic review of the clinical manifestations of cytokine storms and neurotoxicity associated with CAR-T cell therapy reported that the cytokine storm might be a fatal complication of CAR-T cell immunotherapy [45]. Given the lack of a consistent definition for cytokine storms, in 2020, Fajgenbaum et al. [37] proposed 3 diagnostic criteria for cytokine storms: 1) elevated circulating cytokine levels, 2) acute systemic inflammatory symptoms, and 3) secondary organ dysfunction attributable to inflammatory processes. In the same year, during the global respiratory virus pandemic, two seminal studies [46, 47] reported the occurrence of cytokine storms in critically ill patients with severe acute respiratory syndrome coronavirus 2 (SARS-CoV-2) infection, highlighting its contribution to disease progression and mortality. Therefore, cytokine storms have attracted widespread attention, prompting research into their underlying mechanisms, potential therapeutic targets, and clinical management.

## Mechanisms of the cytokine storm

Cytokine storms are a dysregulated immune response characterized by the excessive production and release of proinflammatory cytokines along with the inhibition of the anti-inflammatory response, leading to severe tissue damage and multiorgan failure. This section reviews the key mechanisms underlying its initiation, amplification, and effector phases, drawing from recent evidence obtained in the fields of infectious diseases and immunopathology.

### Triggers of cytokine storms

The onset of a cytokine storm is driven by intricate interactions between the host immune system and danger signals caused by pathogen invasion, tissue injury, or cellular stress. Pathogen-associated molecular patterns (PAMPs), DAMPs, and inflammatory cell death, which activate immune signaling cascades and precipitate a rapid inflammatory response, are central to this process [5, 6].

#### PAMP-pattern-recognition receptors (PRRs) interactions

**Fig. 1:**
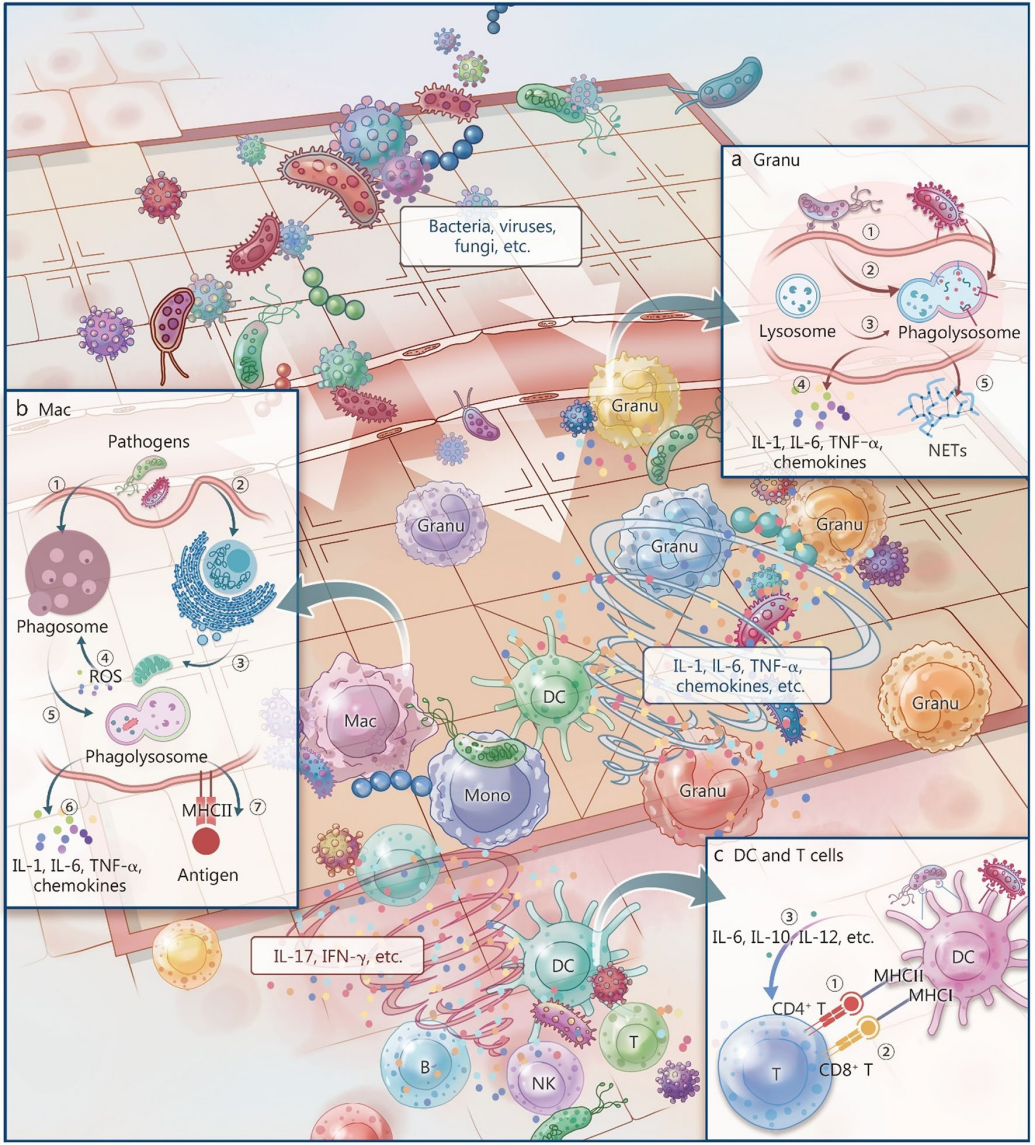
Schematic of cytokine storms initiation in response to infection. When pathogens such as bacteria, viruses, or fungi invade a host and elicit an immune response, inadequate clearance may allow dissemination through the bloodstream. The innate immune system becomes rapidly activated, triggering the release of numerous cytokines from granulocyte (Granu), macrophage (Mac), monocyte (Mono), natural killer (NK) cell, and dendritic cell (DC). For example, **a** Neutrophils eliminate pathogens through phagocytosis and NETosis. ① Neutrophils recognize pathogen-associated molecular patterns via pattern recognition receptors; ② pathogens are engulfed via phagocytosis; ③ phagosomes fuse with lysosomes to form phagolysosomes; ④ cytokines such as interleukin (IL)-1, IL-6, tumor necrosis factor-α (TNF-α), and chemokines are released, amplifying immune signaling; and ⑤ in the presence of an excessive pathogen load, neutrophils undergo NETosis, releasing neutrophil extracellular traps (NETs) composed of DNA and antimicrobial proteins. NETs trap and kill pathogens extracellularly and stimulate adaptive immune cells, promoting immune activation. **b** Mac kills pathogens via oxidative stress. ① Pathogens are internalized into early endosomes; ② phagocytosis induces endoplasmic reticulum (ER) stress; ③ ER stress activates mitochondria, generating reactive oxygen species (ROS); ④ mitochondrial ROS-containing vesicles increase bactericidal activity within phagosomes; ⑤ phagolysosome maturation mediates pathogen degradation; ⑥ cytokines, including IL-1, IL-6, TNF-α, and chemokines are secreted to modulate inflammation and immunity; and ⑦ Mac process and present antigens via major histocompatibility complex (MHC) class II to CD4⁺ T cells, initiating adaptive immunity. **c** Dendritic cells (DCs) activate adaptive immunity. ① DCs present antigens to CD4⁺ T cells via MHC class II; ② exogenous antigens are cross-presented on MHC class I to CD8⁺ T cells; and ③ cytokine secretion guides CD4⁺ T helper cell differentiation, shaping adaptive immune responses. The initial release of cytokines (e.g., IL-1, IL-6, TNF-α, and chemokines) aims to eliminate pathogens but may culminate in a cytokine storm. This process amplifies adaptive immune activation, leading to the production of a secondary wave of cytokines such as IL-17 and interferon-γ (IFN-γ). The host-pathogen interaction resembles a strategic game wherein pathogens breach defenses and immune cells respond to counter the invasion

**Fig. 2:**
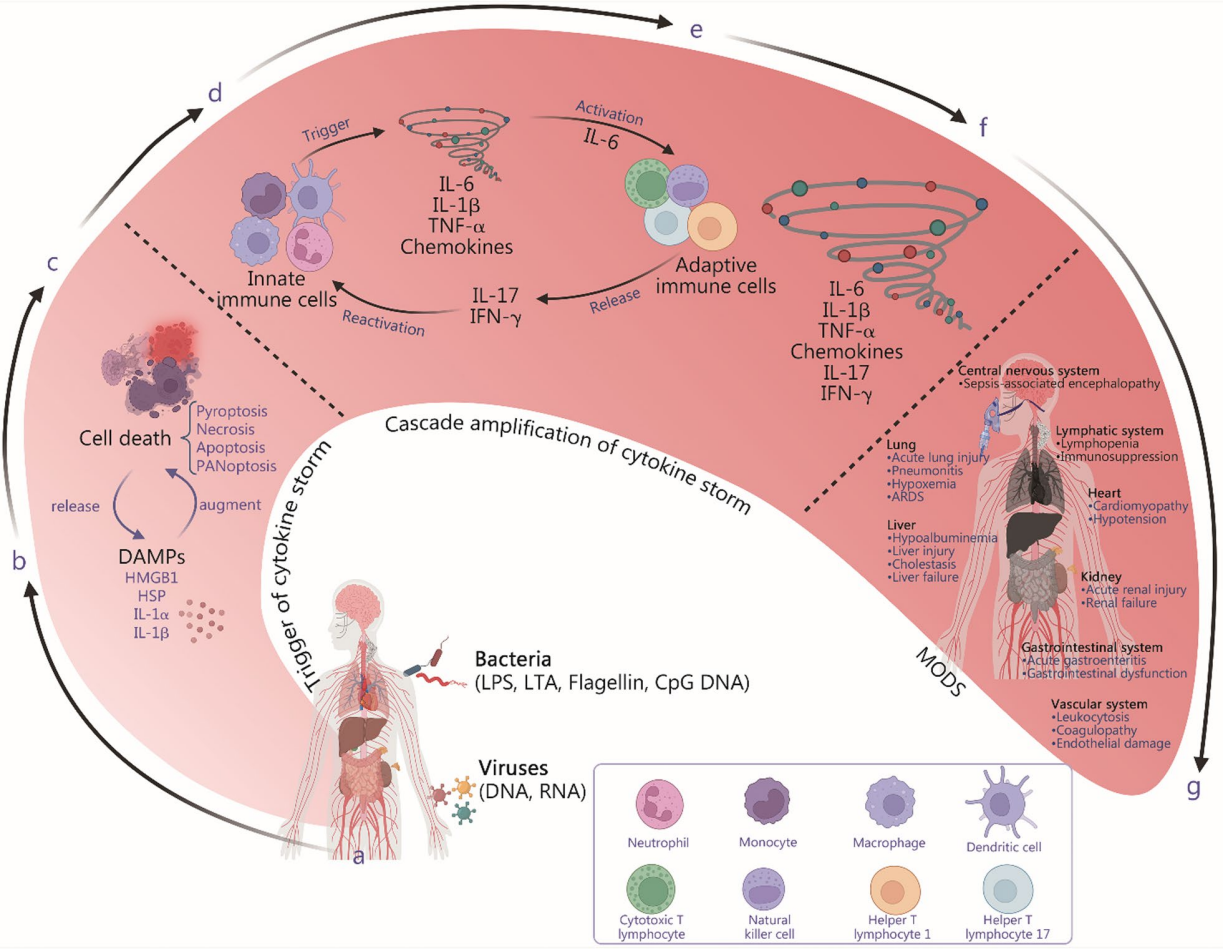
Mechanisms of cytokine storms initiation, amplification, and outcomes. **a** Pathogen invasion: the host is invaded by pathogens such as bacteria, e.g., lipopolysaccharide (LPS), lipoteichoic acid (LTA), flagellin, CpG deoxyribonucleic acid (CpG DNA), viral ribonucleic acid (RNA) and deoxyribonucleic acid (DNA), and fungi. **b** Damage-associated molecular patterns (DAMPs) release: invasion triggers the release of DAMPs, including high mobility group box 1 (HMGB1), heat shock protein (HSP), interferon (IL)-1α, and IL-1β. **c** Cell death and amplification: DAMPs induce cell death, including pyroptosis, necroptosis, apoptosis, and PANoptosis, in immune cells, creating a feedback loop that intensifies the inflammatory response. **d** Innate immune activation: pathogens activate the innate immune system, leading to the reactivation of innate immune cells. **e** Cytokine storms activation: activated innate immune cells initiate a cytokine storm characterized by the release of proinflammatory cytokines such as IL-6, IL-1β, tumor necrosis factor-α (TNF-α), and chemokines. IL-6 further stimulates adaptive immune cells, leading to the secretion of IL-17 and interferon-γ (IFN-γ), which reactivate innate immune cells. **f** Cytokine storms amplification: the cytokine storm is amplified, affecting multiple systems, including the vascular system, lymphatic system, lung, liver, kidney, and gastrointestinal tract, leading to various complications. **g** Multiple organ dysfunction syndrome (MODS): the cytokine storm results in MODS, which is characterized by acute organ injury and dysfunction across different systems, highlighting the severe consequences of unchecked inflammation. ARDS acute respiratory distress syndrome

**Fig. 3:**
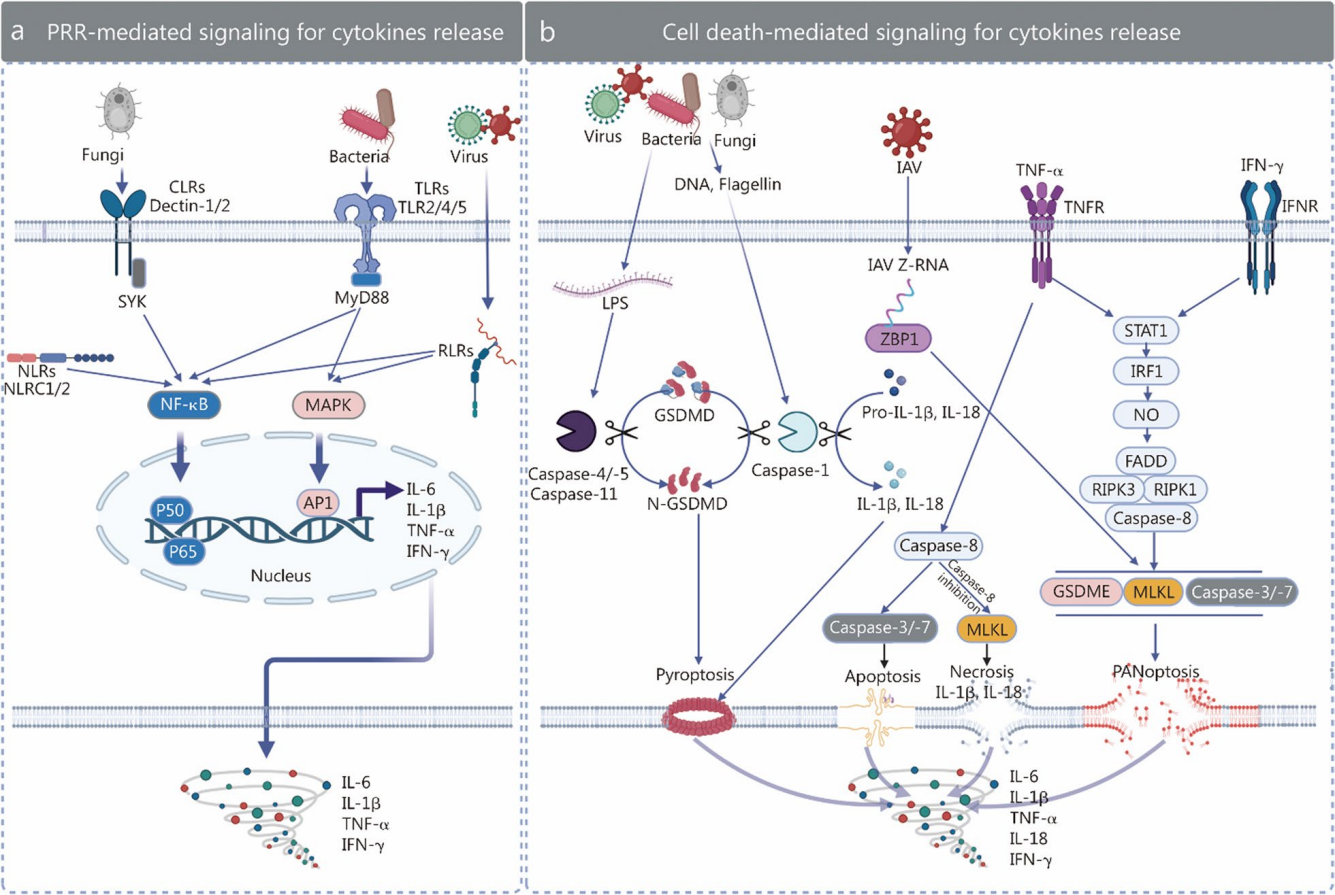
Signaling pathways involved in proinflammatory cytokine production. **a** Pathogen-associated molecular patterns (PAMPs) and damage-associated molecular patterns (DAMPs) are recognized by pattern recognition receptors (PRRs), initiating signaling pathways for proinflammatory cytokine production. Fungi are primarily detected by C-type lectin receptors (CLRs), bacteria are detected by Toll-like receptors (TLRs), viruses are detected by RIG-I-like receptors (RLRs), and NOD-like receptors (NLRs) recognize a variety of danger signals. Upon recognition of these signals, cells activate the nuclear factor kappa-B (NF-κB) or mitogen-activated protein kinase (MAPK) signaling pathways, leading to the transcription of proinflammatory cytokine genes such as interleukin (IL)-6, IL-1β, tumor necrosis factor-α (TNF-α), and interferon-γ (IFN-γ). **b** Proinflammatory cytokine production is also regulated by signaling pathways associated with different forms of cell death, such as pyroptosis, apoptosis, necrosis, and PANoptosis. Pyroptosis is triggered by various danger signals, including pathogens and lipopolysaccharide (LPS). The N-terminal domain of gasdermin D (GSDMD), which is released by cleavage mediated by active caspase-1, caspase-11 (in mice) or caspase-4/-5 (in humans), induces pyroptosis. Caspase-1 also cleaves pro-IL-1β and pro-IL-18 into their active forms, which are released through pyroptotic pores. Apoptosis and necrosis can be induced by the binding of TNF-α to its receptor, tumor necrosis factor receptor (TNFR). Apoptosis is executed by activated caspase-8 and downstream caspases-3/-7, whereas necrosis is induced when caspase-8 is inhibited, activating the Mixed lineage kinase domain-like (MLKL) complex. The activated MLKL complex translocates to the cell membrane and causes necrosis. PANoptosis is induced by the binding of TNF-α and IFN-γ or influenza A virus (IAV) Z-ribonucleic acid (Z-RNA) and Z-deoxyribonucleic acid binding protein 1 (ZBP1) of cells. The signal transducer and activator of transcription (STAT) 1 signaling pathway is activated and ultimately induces PANoptosis through gasdermin E (GSDME), MLKL, and caspase-3/-7, leading to the release of cytokines such as IL-6, IL-1β, TNF-α, IL-18, and IFN-γ. SYK spleen tyrosine kinase, NLRC nucleotide-binding oligomerization domain-like receptor C, AP1 activator protein 1, MyD88 myeloid differentiation primary response 88, TNFR tumor necrosis factor receptor, IFNR interferon receptor, STAT signal transducer and activator of transcription, IRF1 interferon regulatory factor 1, NO nitric oxide, FADD Fas-associated via death domain, RIPK receptor-interacting protein kinase 1

Pathogenic microorganisms possess conserved molecular motifs, termed PAMPs, which are absent in the host and therefore act as "danger" signals. Representative PAMPs include LPS of Gram-negative bacteria, peptidoglycan and lipoteichoic acid of Gram-positive bacteria, flagellin, microbial nucleic acids such as cytosine-phosphate-guanine DNA, double-stranded ribonucleic acid (RNA) and single-stranded RNA, and fungal β-glucans [48]. These motifs are sensed by host PRRs expressed on both immune and non-immune cells [49]. Ligation of PRRs triggers tailored effector programs. Upon recognition of PAMPs, neutrophils eliminate pathogens by phagocytosis or NETosis [9, 50] (Fig. 1a), while macrophages engulf and kill pathogens via oxidative stress [16, 31] (Fig. 1b), and DCs process and present antigens, initiate adaptive immunity (Fig. 1c). Thus, PAMP-PRR engagement is the pivotal ignition switch for immune activation and the inflammatory cascade (Figs. 2a and 3a).

The key families of PRRs include TLRs, NOD-like receptors (NLRs), and retinoic acid-inducible gene I-like receptors (RLRs). TLRs are membrane-bound or endosomal receptors that recognize diverse PAMPs. For example, TLR4 binds LPS, while TLR3, TLR7, TLR8, and TLR9 detect viral nucleic acids [51]. TLR4 signaling activates transcription factors such as NF-κB, MAPK, and IFN regulatory factors through myeloid differentiation primary response 88 (MyD88) or toll/interleukin-1 receptor (TIR) domain-containing adapter inducing IFN-β-dependent pathways, promoting the synthesis of proinflammatory cytokines (e.g., IL-6, IL-1β, and TNF-α) and type I IFNs (IFN-α/-β). NLRs function as cytoplasmic sensors of bacterial components and cellular stress. Notably, NLRP3 assembles inflammasomes that activate caspase-1, which cleaves pro-IL-1β and pro-IL-18 into their bioactive forms [52]. RLRs detect viral RNA in the cytoplasm. Through the adaptor mitochondrial antiviral signaling protein, RLRs signaling activates interferon regulatory factor (IRF) 3/7 and NF-κB, inducing robust type I IFN production and antiviral responses [53].

#### Release and action of DAMPs

In addition to exogenous PAMPs, endogenous danger signals, termed DAMPs or alarmins, are released or exposed upon severe tissue injury, ischemia-reperfusion injury, or cellular stress. DAMPs include high mobility group box 1 (HMGB1), heat shock proteins (HSP), extracellular matrix fragments, adenosine triphosphate, uric acid crystals, mitochondrial deoxyribonucleic acid (DNA), and S100 proteins (Fig. 2b). These proteins are recognized by PRRs (e.g., TLRs and NLRs) and other receptors, such as the receptor for advanced glycation end product, triggering proinflammatory pathways akin to those activated by PAMPs, such as the NF-κB and MAPK pathways, and those involved in inflammasome assembly [48]. In infectious conditions, DAMP release often follows PAMP-induced inflammation and tissue damage, perpetuating immune activation and amplifying the inflammatory response. Even after pathogen clearance, DAMPs can sustain uncontrolled inflammation, contributing to the self-perpetuating nature of cytokine storms [54].

#### Inflammatory cell death induces cytokine storms

In recent years, various modes of cell death, such as gasdermin D-mediated pyroptosis, caspase-3/-7-dependent apoptosis, and mixed lineage kinase domain-like-driven necroptosis, have been reported [55–57]. These distinct forms of cell death collectively facilitate the release of large quantities of cytokines. PANoptosis is a newly identified type of programmed cell death characterized by the simultaneous or concerted activation of key signaling pathways underlying pyroptosis, apoptosis, and necroptosis. Notably, inhibiting only one of these cell death modalities is insufficient to prevent cell death. For instance, in viral pneumonia, PANoptosis can be triggered by the synergistic actions of TNF-α and IFN-γ or activated upon Z-DNA binding protein 1 (ZBP1) recognition of influenza A virus (IAV) [5, 58]. This review discusses cell death phenomena associated with cytokine storms, encompassing three classical forms of cell death, including pyroptosis, apoptosis, and necroptosis, and this unique and novel programmed cell death pathway, PANoptosis (Figs. 2c and 3).

In the early stages of infection, the recognition of PAMPs by PRRs initiates a strong inflammatory response and promotes the release of a large number of cytokines (Fig. 3a) while also triggering inflammatory cell death. For example, some PAMPs, such as Z-RNA derived from IAV, can trigger PANoptosis, and LPS can induce pyroptosis. Subsequently, certain cytokines (such as TNF-α) can induce necroptosis and apoptosis. TNF-α and IFN-γ can also act synergistically to collectively activate PANoptosis (Fig. 3b).

Inflammatory cell death is triggered during the early stages of infection or stress, leading to the release of cellular contents such as cytokines and DAMPs. These molecules further amplify immune activation, promote the development of early cytokine storms, and accelerate tissue damage and organ dysfunction [57].

### Mechanisms underlying the amplification of the cytokine storm

Amplification of the cytokine storm involves the establishment of positive feedback loops and the failure of negative feedback mechanisms, resulting in uncontrolled inflammation. This process is particularly evident in sepsis and other severe infections, where it transitions from localized to systemic responses.

#### Formation of positive feedback loops: from ordered initiation to uncontrolled amplification

The initial wave of amplification is driven by innate immune cells (e.g., neutrophils, macrophages, and DCs) (Fig. 2d). The recognition of PAMPs/DAMPs via PRRs triggers the production of key proinflammatory cytokines such as IL-6, IL-1β, and TNF-α [59, 60] (Fig. 2e). These cytokines act in autocrine and paracrine manners to further activate the NF-κB and MAPK pathways in the cytokine-producing cells, creating a positive feedback loop that increases their own synthesis. Cytokines activate endothelial cells, increasing vascular permeability and upregulating adhesion molecules such as intercellular adhesion molecule-1 (ICAM-1) and vascular cell adhesion molecule-1 (VCAM-1) to recruit more leukocytes to the site of infection/injury [61–63] and stimulate the release of chemokines, which amplify the recruitment and activation of additional innate immune cells [64, 65].

The innate immune response orchestrates the adaptive immune system, leading to a second, more potent wave of amplification [66]. T helper type (Th) 1 cells, which are driven by IL-12 and IFN-γ, are a major source of IFN-γ. IFN-γ strongly synergizes with TNF-α and IL-1β to hyperactivate macrophages, leading to a massive secondary increase in the production of proinflammatory cytokines via the Janus tyrosine kinase/signal transducer and activator of transcription (JAK/STAT1) pathway [67–71]. This process represents a critical positive feedback loop between innate and adaptive immunity. Th17 cells, which are polarized by IL-6 and transforming growth factor-β (TGF-β), produce IL-17A/F [72]. IL-17 acts on stromal and epithelial cells to induce the production of chemokines that are potent recruiters of neutrophils, leading to intense neutrophil inflammation and tissue damage [73] (Fig. 2e).

#### Failure of the negative feedback loop mechanism

Under physiological conditions, the immune system employs tightly regulated amplification mechanisms to eliminate pathogens while minimizing collateral damage. Innate immune amplification is a rapid, broad first response aimed at containing threats and orchestrating the subsequent adaptive response. Adaptive immune amplification, mediated by antigen-specific T and B cells, provides a potent, targeted, and long-lasting response. This process is characterized by precise feedback controls. In the innate immune system, activated macrophages and monocytes produce anti-inflammatory cytokines such as IL-10 and TGF-β to form an early autoregulatory loop [74–76], while myeloid-derived suppressor cells (MDSCs) expand during inflammation and suppress T-cell and natural killer (NK)-cell activity through arginase-1, inducible nitric oxide synthase, and other mechanisms [77]. In the adaptive immune system, regulatory T cells (Tregs) act as master regulators, suppressing effector T-cell proliferation and function via contact-dependent mechanisms and secretion of IL-10 and TGF-β [78]. Certain B-cell subsets, particularly regulatory B cells (Bregs), also contribute to inflammation resolution through IL-10 production [79, 80]. Together, these mechanisms ensure that inflammation resolves after threat clearance and immune homeostasis is maintained [74, 81].

The pathological state of cytokine storms arises when these regulatory mechanisms fail. The initial inflammatory trigger is often too strong or persists because of ineffective pathogen clearance, leading to the loss of negative feedback control, such as IL-10 resistance [75] and Treg exhaustion [82], while positive feedback loops continue unabated. The result is a self-perpetuating, escalating cycle of inflammation that is decoupled from the original stimulus, leading to widespread tissue injury and organ failure.

### Cytokine storms in sepsis

The pathology of cytokine storms occurs when this sophisticated regulatory system is overwhelmed or disrupted. We have clarified the mechanisms of failure, including cytokine resistance, where high levels of TNF-α and other inflammatory mediators can induce resistance to IL-10 [19, 75], the functional exhaustion of regulatory cells, such as Tregs and MDSCs, which can lose their suppressive capacity in intense inflammatory milieus [77, 78, 82], and extensive lymphocyte apoptosis that depletes the cells capable of terminating the response [78]. This failure to apply the "brakes" is what allows the proinflammatory "accelerator" to remain floored, leading to uncontrolled amplification and collateral tissue damage (Fig. 2f).

#### Systemic disease

The cytokine storm exerts deleterious effects through widespread organ damage and systemic dysfunction, which are mediated by proinflammatory cytokines. These mechanisms are interconnected, leading to MODS and high mortality in patients with sepsis and viral infection (Fig. 2g).

Pathogens and their components directly induce tissue damage through multiple mechanisms. For instance, Gram-negative bacterial LPS disrupts endothelial tight junctions via TLR4-mediated endothelial activation, leading to vascular leakage and microcirculatory dysfunction [27, 83], whereas the SARS-CoV-2 spike protein binds to ACE2 on alveolar epithelial cells to trigger apoptotic cell death and impair gas exchange [16]. Moreover, persistent bacteremia/viremia sustains a feed-forward loop, with pathogen replication releasing additional PAMPs/DAMPs to amplify the cytokine storm, which subsequently compromises epithelial/endothelial barriers and facilitates pathogen dissemination [84]. Clinical evidence supports this synergy. In septic patients, elevated LPS levels correlate with both increased IL-6 concentrations and worse severity of acute kidney injury (AKI), and targeted antibacterial therapy (e.g., carbapenems) reduces both bacteremia and cytokine-driven organ damage. Similarly, in patients with viral pneumonia, the viral load correlates with ARDS progression, and antiviral agents (e.g., remdesivir) mitigate lung injury by reducing viremia and subsequent cytokine release [46]. Thus, organ injury during cytokine storms is driven not solely by proinflammatory responses but also by reciprocal interactions between pathogens (or their products) and dysregulated immunity of which are critical therapeutic targets.

##### Direct cytokine-induced toxicity and indirect hemodynamic effects (septic shock)

High circulating levels of cytokines such as TNF-α, IL-1β, and IFN-γ can directly bind to receptors on parenchymal cells (e.g., cardiomyocytes, hepatocytes, and renal tubular cells), triggering intracellular proapoptotic or proinflammatory pathways and metabolically disruptive signals. This direct toxicity results in specific forms of cellular dysfunction such as impaired cardiac contractility, reduced hepatic gluconeogenesis, and the loss of epithelial barrier integrity [85], culminating in organ damage and conditions such as ARDS [5, 58].

Concurrently, the systemic inflammatory response causes indirect, widespread damage primarily through septic shock. This state is defined by severe vascular dysfunction, resulting in profound vasodilation, hypotension, and maldistribution of blood flow, which leads to global tissue hypoperfusion. The resulting distributive shock means that vital organs are starved of oxygen and nutrients. This effect is often compounded by DIC, where microthrombus formation obstructs the microcirculatory flow, causing further ischemic injury [83].

##### Acute lung injury (ALI)/ARDS

The lungs are highly susceptible to cytokine storms, which can induce ALI/ARDS through macrophage and neutrophil recruitment, leading to the release of proinflammatory cytokines (TNF-α and IL-6) and surfactant damage [86]. This process results in alveolar exudation, impaired gas exchange, and hypoxemia, which are exacerbated by JAK/STAT signaling [87]. In patients with sepsis, elevated cytokine levels in bronchoalveolar lavage fluid correlate with increased ARDS severity and mortality [88].

##### Vascular endothelial injury and microcirculation dysfunction

Cytokines such as TNF-α and IL-1β target endothelial cells, upregulating the expression of adhesion molecules (E-selectin and VCAM-1) and disrupting tight junctions, which increase vascular permeability and promote edema [89]. Additionally, they induce tissue factor expression and inhibit anticoagulant mechanisms, triggering DIC and microthrombosis, compromising tissue perfusion and contributing to organ failure [83]. Microcirculatory disturbances are strongly linked to adverse outcomes in patients with severe infections [90].

*AKI*. Cytokine storms contribute to AKI via renal hypoperfusion, direct cytokine-induced toxicity (e.g., TNF-α and IL-1β), and impaired microcirculation [91]. In addition to the development of microthrombi, inflammatory and apoptotic signaling reduce renal function, causing electrolyte imbalances and waste accumulation. Biomarkers such as IL-18, C-C motif chemokine ligand 2 (CCL2), and CCL14 are indicators of AKI progression [92].

*Myocardial dysfunction*. Cardiac impairment in patients with sepsis involves cytokines (e.g., TNF-α and IL-6) that disrupt calcium homeostasis, mitochondrial function, and contractility while inducing nitric oxide overproduction, which reduces myocardial responsiveness. Endothelial injury and microthrombi exacerbate ischemia, leading to decreased cardiac output and a poor prognosis [93].

*Liver dysfunction*. In the liver, which is both a source and a target of inflammation, hepatocyte injury and apoptosis induced by TNF-α occur, with Kupffer cells amplifying cytokine release [94]. Microcirculatory disturbances and cholestasis contribute to elevated liver enzyme levels, reduced liver cell proliferation, and potential liver failure [95].

*Central nervous system dysfunction*. The brain is highly protected by autoregulatory mechanisms. Neurological failure (sepsis-associated encephalopathy) is typically a late and ominous sign, indicating a complete loss of cardiovascular homeostasis and the failure of cerebral autoregulation [96]. Sepsis-associated encephalopathy is a severe complication of sepsis characterized by diffuse brain dysfunction without direct central nervous system infection. Systemic inflammation plays a central role in its pathogenesis, with proinflammatory cytokines crossing the compromised blood-brain barrier and activating microglia, leading to neuroinflammation, oxidative stress, and neuronal damage [97, 98]. These processes contribute to acute cognitive impairments, including delirium, and are associated with long-term cognitive deficits in survivors [99, 100].

*Gastrointestinal dysfunction*. The gut is not only a target organ in individuals with MODS but also a critical amplifier that drives systemic inflammation and the cytokine storm [101–103]. While intestinal barrier breakdown occurs early and may fuel a cytokine storm via bacterial translocation, overt clinical failure (e.g., paralytic ileus and stress ulcers) often occurs later [104].

#### Immunosuppression and secondary infections

After a cytokine storm, a compensatory anti-inflammatory response syndrome occurs, increasing the risk of secondary infection. Pathophysiological mechanisms include lymphocyte apoptosis, the excessive production of anti-inflammatory cytokines, functional paralysis of immune cells, and T lymphocyte exhaustion, which collectively decrease the pathogen clearance capacity and increase susceptibility to nosocomial infections [78]. Epidemiological evidence indicates that patients who have recovered from sepsis and other severe infections face a substantial risk of secondary bacterial or fungal infections, representing a significant contributor to elevated long-term mortality rates [105, 106].

Immune suppression can occur both after a cytokine storm (sequential) and, in some cases, in parallel, with two leading hypotheses proposed to explain this process. The sequential hypothesis is dominant in most severe infections and sepsis. Early cytokine storms trigger compensatory anti-inflammatory response syndrome (CARS) as a feedback mechanism: prolonged increases in TNF-α and IL-6 levels exhaust T cells (via apoptosis) and increase the number of MDSCs, leading to immune paralysis after the resolution of the cytokine storm (7–10 d) after its onset [107]. The parallel phenomenon is observed in patients with heterogeneous conditions such as viral pneumonia and trauma. It involves simultaneous hyperinflammation and local immunosuppression. For instance, patients with viral pneumonia may exhibit a systemic increase in IL-6 levels alongside lung-localized T-cell exhaustion, the latter of which is characterized by programmed cell death protein-1 (PD-1) upregulation and driven by viral persistence [106].

Ultimately, the cytokine storm exemplifies a "pyrrhic victory" for the host. Although the initial hyperactive immune response aims to eradicate pathogens, the subsequent cascade of events results in devastating multiorgan damage and systemic failure. The body’s own defense mechanisms, which are intended for protection, become agents of self-destruction, leaving the host severely debilitated even if the initial infection is cleared [78]. This finding highlights the urgent need for therapeutic interventions that can effectively modulate the immune response, preventing the escalation from protective immunity to uncontrolled inflammation and its dire consequences [105–107].

## Therapeutic strategies for the cytokine storm

International guidance for managing cytokine storms is etiology-specific, and a “one-size-fits-all” protocol does not exist. Below, the main categories are summarized along with the most influential consensus statements. The Surviving Sepsis Campaign 2021 did not recommend any cytokine inhibitors. Hydrocortisone (200 mg/d) is advised only for patients with shock requiring treatment with vasopressors [105]. For CAR-T cell-related cytokine storms, the American Society for Transplantation and Cellular Therapy 2020 consensus statement divides severity by hypotension, hypoxia, and organ dysfunction and then recommends stepwise IL-6 blockade (tocilizumab ± corticosteroids) as a first-line treatment for grade ≥ 2 disease, with siltuximab or anakinra as alternatives [106, 108]. For patients with severe viral pneumonia and hyperinflammation, the living guideline of the World Health Organization (WHO) 2022 stratifies patients by the oxygen requirement for treatment with an IL-6 blocker (tocilizumab or sarilumab) plus systemic corticosteroids if the C-reactive protein (CRP) concentration is ≥ 75 mg/L or the PaO_2_/FiO_2_ is < 300 mmHg. JAK inhibitors (baricitinib or tofacitinib) can be added when baricitinib is available [74]. For the treatment of idiopathic/secondary HLH and MAS, the Histiocyte Society HLH-2004 statement indicates that dexamethasone with etoposide remains the main approach [109]. For patients with MAS, 2–8 mg/(kg‧d) anakinra can be started empirically even before genetic confirmation because rapid IL-1 suppression reduces the intensive care unit (ICU) stay [110]. With respect to viral hemorrhagic fever (Ebola and Lassa), the WHO 2018 Ebola interim protocol lists cytokine storm-like presentations as the “critical phase” and recommends supportive care only. No biologics are advised because of the theoretical risk of viral reactivation [111]. In conclusion, the recommendations differ across diseases. IL-6 blockade is the standard treatment for CAR-T and viral pneumonia. IL-1 blockade is favored for MAS.

In general, the currently employed therapeutic approaches for cytokine storms aim to mitigate hyperinflammatory responses, restore immune homeostasis, and prevent secondary infections [105]. This section outlines strategies targeting excessive inflammation and immunosuppression, highlighting their mechanisms, clinical evidence, and challenges. In addition to conducting systematic searches in traditional databases such as PubMed, Embase, and Web of Science, we also performed a systematic search on the World Health Organization International Clinical Trials Registry Platform (https://trialsearch.who.int/Default.aspx) using “cytokine storm” and commonly used therapeutic drugs as keywords to comprehensively understand the current research landscape of therapeutic agents for cytokine storms. This search yielded 8915 ongoing studies. After a careful review of the registration information for each study, we ultimately identified 104 ongoing randomized controlled trials (RCTs). Among these, 6 were multinational collaborative studies, 5 focused on Coronavirus Disease 2019 (COVID-19), and 1 targeted candidemia. The drugs under investigation included remdesivir, IFNγ-1b, emapalumab, anakinra, canakinumab, glucocorticoids, and ruxolitinib. The detailed search strategy and a list of basic information for the ongoing RCTs are provided in the Additional file 1.

### Therapies targeting excessive inflammation

Excessive inflammation is a primary cause of early organ damage and mortality. Therapeutic strategies focus on dampening proinflammatory cascades while preserving protective immune functions.

#### Cytokine antagonists

IL-1 antagonists have garnered attention as therapeutic options for managing cytokine storms, especially in the context of viral infection. IL-1 is a key mediator of the inflammatory response, and its overproduction is associated with severe inflammatory conditions [112]. Anakinra, a recombinant IL-1 receptor antagonist, has shown efficacy in reducing inflammation and improving clinical outcomes in patients experiencing severe viral infection-related cytokine storms [113]. Moreover, its safety profile is favorable, with fewer adverse effects than systemic corticosteroids, making it a suitable option for patients at risk of secondary infections due to immunosuppression [113]. The timing of anakinra administration is a critical determinant of treatment efficacy. Initiating treatment during the early stages of the cytokine storm may maximize its benefits, preventing progression to severe respiratory failure [114]. As researchers continue to elucidate the roles of IL-1 in viral infection and other hyperinflammatory conditions, anakinra and similar agents may become integral components of treatment protocols aimed at controlling excessive inflammatory responses.

IL-6, a pleiotropic cytokine, plays complex and critical roles in the immune response through two distinct signaling pathways [115]. The classic signaling pathway mediates protective immune functions upon the binding of IL-6 to the membrane-bound IL-6 receptor (IL-6R) and the gp130 coreceptor, thereby recruiting and activating immune cells and promoting pathogen clearance, which are crucial for host defense against infection [68]. In contrast, the trans-signaling pathway involves the binding of IL-6 to soluble IL-6R, resulting in the formation of a complex that can activate cells expressing only gp130, leading to amplified inflammation and potential tissue damage [68]. Overactivation of the trans-signaling pathway is considered a key driver of inflammatory cytokine storms and tissue pathology in certain pathological conditions, particularly severe infections [68]. Nonselective inhibitors such as the anti‑IL‑6R antibody tocilizumab block both pathways. Therefore, when these nonselective inhibitors suppress pathogenic inflammation, they may also impair the protective functions of classic signaling (for example, compromising bacterial clearance in patients with sepsis), which likely contributes to variable or disappointing clinical results across different infectious etiologies [67]. In contrast, selectively targeting IL‑6 trans‑signaling, for example, with agents modeled on soluble glycoprotein 130 Fc fusion protein signal transducer and activator of transcription (sgp130Fc), could inhibit the deleterious, inflammation‑amplifying arm while preserving host defense mediated by the classic signaling pathway, a precision strategy that may improve therapeutic efficacy against inflammatory diseases and represents a promising treatment for complex conditions such as sepsis [115]. Nevertheless, rigorous preclinical and clinical validation in the context of disease heterogeneity is still needed.

#### Triggering receptor expressed on myeloid cells-1 (TREM-1) inhibitors

The role of triggering receptor expressed on TREM-1 in sepsis can be summarized as an "inflammatory amplifier". Upon activation by pathogens or danger signals, it significantly amplifies the cascade of proinflammatory cytokines such as TNF-α, IL-1β, and IL-8 by synergizing with Toll-like receptor signaling, thereby accelerating systemic inflammatory cytokine storms and multiorgan damage [116]. Its soluble form, soluble TREM-1 (sTREM-1), serves as a biomarker for early diagnosis and prognostic assessments, while blocking TREM-1 signaling has been shown to reduce inflammation and prolong the survival of animal models, making it a potential target for precision intervention [117]. TREM-1 is an activating receptor on neutrophils and monocytes that triggers inflammatory responses to pathogens via DNAX-activating protein of 12 kD (DAP12) [118]. Nangibotide is a TREM-1 inhibitor that acts as a decoy receptor for TREM-1 ligands, thereby dampening the excessive inflammation driven by the activation of the TREM-1 pathway. Clinical trials (Phase 2a and 2b) have confirmed its safety and suggested its potential efficacy in patients with high sTREM-1 levels, supporting further Phase 3 investigations in this targeted population [119, 120].

#### C5a inhibitors

In a foundational study, Huber-Lang et al. [121] reported that in the absence of complement component 3 (C3), thrombin can act as a functional C5 convertase, generating C5a and driving inflammatory lung injury independent of traditional complement pathways. This study revealed novel crosstalk between the coagulation and complement systems. C5a was therapeutically targeted with vilobelimab, a monoclonal antibody, in critically ill COVID-19 patients requiring invasive mechanical ventilation [122]. The Phase 3 PANAMO trial demonstrated that compared with the placebo, vilobelimab significantly reduced 28-day and 60-day all-cause mortality without increasing the infection risk, supporting the role of C5a as a key mediator in severe inflammatory respiratory syndromes. Together, these findings indicate a novel pathological mechanism and its clinical application in modulating complement-driven inflammation.

#### Adrenomedullin (ADM) inhibitors

ADM is a multifunctional peptide hormone that plays key roles in regulating vascular tone, endothelial barrier integrity, and the inflammatory response [123]. In patients with sepsis, ADM levels increase significantly and are correlated with disease severity and mortality. The non-neutralizing anti-ADM antibody adrecizumab selectively binds ADM, increasing its plasma concentration by reducing proteolytic degradation and redistributing it from the interstitium to the bloodstream. This shift enhances endothelial barrier function while minimizing vasodilation, leading to improved hemodynamics, reduced organ damage, and increased survival of animal models of sepsis [124, 125]. Clinical trials, such as the Phase 2a AdrenOSS-2 study, have shown that adrecizumab is safe and well-tolerated in patients with septic shock presenting elevated bio-ADM levels. Although not powered for efficacy, the secondary outcomes suggested potential benefits in organ function and reduced mortality [126].

#### JAK inhibitors

JAK inhibitors are a class of emerging immunomodulatory agents that regulate immune responses by targeting the JAK signaling pathway, thereby blocking cytokine signal transduction [127]. JAK inhibitors, such as tofacitinib, baricitinib, and ruxolitinib, selectively inhibit different JAK subtypes and effectively reduce the expression of various cytokines, such as IL-6, which plays a crucial role in sepsis and severe infections [127]. JAK inhibitors function as a "double-edged sword" in the treatment of sepsis and severe infections. On the one hand, they can suppress excessive inflammation, benefiting critically ill patients with infectious diseases. On the other hand, they may weaken the host’s anti-infectious activity, increasing the risk of certain infections [128]. For example, in several sepsis models [e.g., cecum ligation and puncture (CLP) and LPS-induced models], the use of JAK inhibitors can reduce proinflammatory cytokine levels, alleviate organ damage, and increase survival rates in the short term [129, 130]. In the clinical context of COVID-19, in particular, baricitinib has been shown to inhibit cytokine storms and reduce mortality in severely ill patients [131]. However, in some infection models, JAK inhibitors impair host defense mechanisms, increasing susceptibility to bacterial and viral infections. For example, in a model of *Staphylococcus aureus*-induced septic arthritis, mice treated with tofacitinib exhibited more severe joint erosion [130]. In individuals with herpes simplex encephalitis, viral clearance was impaired [131, 132]. Therefore, more clinical studies are needed in the future to further explore the mechanisms of action of JAK inhibitors in different infectious diseases.

#### Caspase inhibitors

Targeting caspases to block pyroptosis and inflammation has shown promise in preclinical models. For example, the caspase-1 inhibitor AC-YVAD-CMK suppresses NOD-like receptor protein 1 (NLRP1) inflammasome activation and decreases IL-1β and IL-18 expression, alleviating sepsis-induced AKI in mice [133]. Nisoldipine, a calcium channel blocker, also inhibits the activation of inflammatory caspases (1 and 4/11), prolonging the survival of septic mice [134]. However, pharmacological limitations (e.g., poor stability) hinder its clinical translation.

#### Glucocorticoids (GCs)

GCs have been historically used as broad-spectrum immunomodulatory agents in patients with severe infections such as sepsis. They exert potent anti-inflammatory effects by suppressing proinflammatory cytokine production [135]. However, the efficacy of GCs against severe infections remains controversial. While some clinical trials suggest potential benefits in reducing mortality in patients with septic shock and relative adrenal insufficiency, other studies have shown opposite effects [135–137]. More importantly, the dual mechanism of action of GCs is at the heart of the controversy surrounding their efficacy. On the one hand, in the early stages of infection, the potent anti-inflammatory effects of GCs may inhibit excessive immune responses and reduce tissue damage [138]. On the other hand, GCs may also suppress immune responses crucial for pathogen clearance [139]. Furthermore, GCs can affect glucose metabolism, which may have adverse effects on patients during infection [140].

The impacts of GCs on the treatment response in different infection sources (e.g., respiratory versus nonrespiratory infections), pathogen spectra, and underlying host conditions have not been clearly defined. Combination therapy with hydrocortisone and fludrocortisone has shown additional benefit in some trials, but other studies have failed to consistently reproduce these results [141, 142]. Currently, a consensus on the optimal GC regimen for sepsis treatment, including the dosage, initiation timing, and treatment duration, is lacking [143, 144]. Evidence regarding the efficacy and safety of GCs in specific subpopulations, such as pediatric patients, patients with non-COVID-19 viral sepsis, individuals with central nervous system comorbidities, or patients with postoperative sepsis, remains insufficient [143–145]. The development of individualized, precision-stratified dosing strategies is still in the exploratory stage. A deeper understanding of the complex regulatory roles of GCs at different cellular and molecular levels is needed to better identify patient populations that may truly benefit.

#### Blood purification therapies

Blood purification represents an emerging therapeutic strategy for managing cytokine storms, excessive immune responses frequently associated with critical illnesses such as sepsis [146]. This approach aims to mitigate excessive inflammation by directly removing inflammatory mediators, including cytokines and pathogens, from the bloodstream using techniques such as hemoperfusion and plasmapheresis [147]. Several specific devices are under investigation for this purpose. The Seraph® 100 Pathogen Adsorption Column was safe in a first-in-human trial involving hemodialysis patients [148], and its efficacy in patients with sepsis is currently being evaluated in multicenter trials (NCT04260789). CytoSorb®, a cytokine adsorber, showed modest clearance of IL-6 in septic patients with ALI, although it did not significantly impact mortality or the ventilation duration in a previous study [149]. However, retrospective data suggest that compared with standard renal replacement therapy, it may increase 28-day survival in patients with septic shock [150]. The oXiris® filter combines cytokine adsorption, endotoxin removal, and renal support functionalities. Studies indicate that it effectively reduces the levels of key cytokines (IL-6, TNF-α, and IFN-γ), improves hemodynamic stability, and decreases lactate levels in patients with septic shock [151, 152].

### Therapies targeting immunosuppression

This section outlines therapeutic approaches aimed at reversing immune suppression, particularly in patients with conditions such as sepsis and severe infection, which are characterized by lymphopenia, cytokine depletion, and dysregulated immune responses. These strategies focus on restoring immune homeostasis, enhancing immune cell function, and re-establishing cytokine production.

#### Cytokine-based immunostimulation

Recombinant IL-7 (rIL-7) shows promise for reversing lymphopenia, a hallmark of immune suppression. By promoting lymphocyte proliferation and homeostasis, rIL-7 can restore immune function. Early clinical trials in septic patients with lymphopenia have shown that rIL-7 increases circulating CD4^+^ and CD8^+^ T-cell counts, potentially reducing susceptibility to secondary infections [153]. A recent clinical trial in critically ill patients with viral infection revealed that rIL-7 administration increased lymphocyte counts and ameliorated secondary infections [154]. These studies suggest that rIL-7 can both reconstitute immune cell populations and mitigate the risks associated with immunosuppression. Furthermore, rIL-7 has a favorable safety profile [154].

Granulocyte-macrophage colony-stimulating factor (GM-CSF) is essential for myeloid cell activation and host defense, but its therapeutic application in severe infections such as sepsis and severe viral infection is complex because of its dual roles in both driving and resolving inflammation [155]. Its most promising use is reversing sepsis-induced immunoparalysis. Clinical studies, particularly with sargramostim (rhGM-CSF), have demonstrated its efficacy in restoring immune competence by increasing monocyte human leukocyte antigen-DR isotype (HLA-DR) expression, reducing the number of MDSCs, ultimately decreasing secondary infections and prolonging survival [156]. In viral infection, the optimal approach is more nuanced. Early administration may increase lung defenses, whereas later intervention may benefit from GM-CSF blockade to mitigate cytokine storms [157]. Crucially, the therapeutic efficacy of both administration and inhibition strategies is dependent on precise patient selection, potentially guided by biomarkers [e.g., monocyte human leukocyte antigen-DR (mHLA-DR) or MDSC levels], as well as optimized timing and dosing to re-establish immune homeostasis without inducing excessive inflammation.

IFN-γ holds therapeutic promise for managing cytokine storms, but its clinical utility remains under investigation. Preclinical studies and some clinical data suggest that IFN-γ can modulate the hyperinflammatory response characteristic of cytokine storms by promoting Th1 differentiation, increasing cytotoxic T-cell and NK cell activities, and directly inhibiting viral replication [158–160]. While some studies have reported reduced proinflammatory cytokine levels and improved clinical outcomes following IFN-γ treatment, particularly under conditions such as severe viral infection, the results have been inconsistent [159, 160]. Challenges include the potential for IFN-γ itself to induce inflammation and the complexity of cytokine storm pathophysiology, which may require combination therapies for optimal efficacy. Ongoing research is exploring the use of IFN-γ in combination with other immunomodulatory agents and is focused on identifying the optimal dosing strategies and patient populations most likely to benefit. Further clinical trials are needed to definitively establish the role of IFN-γ in managing cytokine storms.

#### Immune checkpoint inhibition

Anti-PD-1 therapy, a cornerstone of cancer treatment, is being explored as a potential strategy to reverse immune suppression in patients with severe infections characterized by cytokine storms and T lymphocyte exhaustion. By blocking the PD-1 checkpoint, this therapy reinvigorates exhausted T lymphocytes, enhancing their cytotoxic function and reducing immune tolerance [161]. In individuals with viral infections, anti-PD-1 therapy has shown promise in restoring T lymphocyte function, mitigating hyperinflammation, and improving clinical outcomes, including reducing mortality [162]. However, careful patient selection and precise timing of drug administration are crucial, as anti-PD-1 therapy can exacerbate cytokine storms in some individuals [163]. While the PD-1/PD-L1 pathway has been implicated in the pathogenesis of sepsis-induced immune paralysis in preclinical models, its clinical efficacy in sepsis patients requires further investigation [164].

#### Immune cell-targeted therapies

Immune cell-targeting therapies are emerging as a promising strategy for immunomodulation in severe infections. A preclinical study has demonstrated that nanotherapies reduce the number of proinflammatory M1 macrophages while increasing the number of anti-inflammatory M2 macrophages, mitigating cytokine storms in models of severe infections, which facilitates the subsidence of inflammation [165]. An off-the-shelf apoptotic cell therapy has been designed to reprogram macrophages and restore immune balance [166], and a Phase 1b trial in septic patients reported 100% survival and significant reductions in the levels of proinflammatory cytokines (TNF-α, IL-6, IL-1β, IL-18, and IFN-γ), along with shorter hospital stays [167]. However, further research is necessary to confirm the clinical efficacy and define patient selection criteria.

Mesenchymal stem cells (MSCs) modulate immune responses by balancing pro- and anti-inflammatory cytokines and promoting tissue repair. A Phase 1 trial of adipose-derived MSCs in endotoxemia patients revealed their safety, with high doses eliciting mixed inflammatory and coagulant effects [168].

### Therapeutic strategies targeting the host response

In recent years, researchers have increasingly recognized the importance of the host’s response to cytokine storms in disease progression and have begun to explore ways to improve patient prognosis by modulating factors such as the host’s nutritional status, metabolic rate, and body temperature.

#### Role of nutritional support in modulating the inflammatory response

Nutritional support is essential in the management of septic and critically ill patients with severe infections [169]. The timing, route, and dosage of nutrition significantly affect patient outcomes. Initiating nutritional support early (within 48 h of admission) is specifically linked to lower rates of organ dysfunction and infections, as well as better nutritional and immune status [170]. Furthermore, early enteral nutrition (EEN) helps preserve gut barrier integrity and enhance mucosal immunity, establishing it as the recommended first-line strategy in clinical practice [171]. However, recent large-scale studies have shown mixed results, with some reporting no significant reduction in mortality from earlier initiation of enteral nutrition [172, 173]. This discrepancy has prompted calls for larger multicenter trials to confirm the findings and to identify patient subgroups that derive the greatest benefit from EEN. In terms of dosing, excessive nutrition imposes a metabolic burden and leads to hyperglycemia, dyslipidemia, and an elevated respiratory quotient, potentially impairing immune function and recovery [170]. Therefore, nutrition should balance metabolic tolerance with actual energy expenditure to avoid overfeeding. Contemporary guidelines emphasize the dynamic adjustment of nutrient doses according to the patient’s metabolic state, inflammatory response, and clinical trajectory [170, 174]. In practice, gradual escalation is advised, starting at approximately 60% of the target energy and progressing to full nutrition [170, 174]. However, evidence on optimal immunonutrition regimens remains limited, and high-quality RCTs are needed to clarify their efficacy and safety.

#### Role of metabolic rate regulation in cytokine storms

Infection and inflammation trigger the dramatic remodeling of energy metabolism in the body, and this metabolic disturbance is closely related to the occurrence and maintenance of cytokine storms [175]. In the early stages of sepsis, the body typically exhibits a hypermetabolic state, with increased glycolysis and lactate production [176]. Immune cells, especially macrophages, undergo metabolic reprogramming upon activation, shifting to glycolysis for energy to support their function [177]. However, long-term metabolic imbalances, such as mitochondrial dysfunction and increased oxidative stress, may further drive the progression of chronic inflammation and cytokine storms [177]. Inhibiting glycolysis may be a potential therapeutic strategy. Studies have shown that metformin, as a glycolysis inhibitor, can inhibit macrophage activation and cytokine release [178–180]. Another leading example is the metabolism-centered perspective on sepsis pathogenesis and management [181–183]. This theoretical framework critically highlights the central role of an essential fatty acid (EFA) metabolic disruption in driving sepsis-associated immune dysregulation. A synergistic treatment strategy involving combined supplementation with EFAs, vitamins, and anti-inflammatory agents is proposed [181]. This approach may provide a novel biological foundation and open new directions for clinical translation in the treatment of sepsis. However, clinical studies targeting metabolic pathways are still limited, and further research is needed.

#### Clinical application of body temperature regulation to treat cytokine storms

Body temperature is an adaptive response to infection. A moderate fever response is considered an inherent mechanism of host resistance to infection, which can increase the activity of certain immune cells (such as T cells and NK cells) and may inhibit the growth of pathogens [184]. However, extreme temperatures, both high (hyperthermia, e.g., above 41 °C) and low (hypothermia), are dangerous in patients with sepsis. Hyperthermia can damage cells and deplete resources, whereas hypothermia often signifies a failing immune response and worse outcomes [184, 185]. Targeted temperature management (TTM), which involves controlling body temperature within a specific range through medication or physical cooling, has been widely used in clinical practice. Nevertheless, robust clinical evidence supporting the use of TTM to treat severe infections remains elusive, with conflicting findings from animal studies [184, 185]. Notably, while mild hypothermia (33.0 ± 0.5) °C reduced serum TNF-α levels and myocardial inflammation in a rat model, cold environments can impair pathogen clearance by dose-dependently decreasing neutrophil chemotaxis and macrophage-mediated phagocytosis [186, 187]. Heterogeneity in TTM outcomes is likely attributable to variations in temperature settings (32–34 °C vs. 36 °C) and intervention durations (24 h vs. 72 h) [186]. High-quality RCTs are needed to determine the efficacy of TTM for severe infections and to define optimal therapeutic thresholds and durations.

### Managing the timing and stratification of malignant inflammation and CARS

Clinical observations consistently reveal a temporal shift from hyperinflammation to CARS within the first 7–10 d of critical illness [4, 107]. Administering additional IL-6 or JAK inhibitors after patients have entered CARS may exacerbate immunoparalysis and increase the secondary infection risk [188], whereas the premature delivery of rIL-7 or GM-CSF during the cytokine storm peak can theoretically fuel hyperinflammation [153]. Pragmatic strategies are emerging to correctly target the “immune-switch window”.

#### Dynamic division of time windows and adaptation of therapeutic strategies

This temporal model of sepsis immunotherapy outlines a dynamic strategy across 3 phases. The initial hyperinflammatory phase (0–3 d), marked by elevated TNF-α, IL-6, and CRP levels, focuses on the precise inhibition of hyperinflammation while avoiding potent immunosuppressants to prevent early CARS. For example, in patients with septic shock requiring vasoactive drugs, the initiation of low-dose hydrocortisone (200 mg/d) within 24 h of diagnosis is recommended [189, 190]. This phase is followed by an immune imbalance transition phase (3–7 d), in which decreasing levels of proinflammatory cytokines coincide with impaired cellular immunity (e.g., low monocyte HLA-DR levels), necessitating bidirectional regulation [78]. For patients with residual inflammation, IL-1β inhibitor (e.g., canakinumab) can be continued [191]. Additionally, for patients with a tendency to develop CARS (e.g., lymphocyte count < 0.8 × 10^9^/L), low-dose rIL-7 should be initiated simultaneously to avoid immune paralysis [154]. Finally, in the established CARS phase (beyond 7 d), the focus should shift to immune restoration using agents such as GM-CSF or PD-1 inhibitors, to strictly avoid cytokine antagonists and emphasize infection surveillance to prevent inflammatory rebound. For example, for patients with persistent lymphopenia count (< 0.8 × 10^9^/L), GM-CSF or PD-1 inhibitors are preferred [192]. Moreover, blood cultures should be performed to monitor secondary infections and avoid the excessive rebound of immune activation.

#### Biomarker panel for patient stratification

This multidimensional biomarker panel integrates key indicators of inflammatory activity, immune function, and organ injury to construct a quantifiable and clinically actionable framework for addressing the heterogeneity of cytokine storm syndrome. It links specific biomarker thresholds to distinct pathological states, including hyperinflammation and CARS, as well as to organ dysfunction risks, thus enabling a shift from empirical treatment toward phenotype-guided interventions. Inflammatory activity is assessed using IL-6 (cut-off of 100 pg/ml for hyperinflammation resolution), CRP (dynamic change > 50% within 24 h, indicating inflammatory fluctuation), and procalcitonin (> 2 ng/ml, suggesting bacterial infection-related inflammation). Immune function is evaluated by measuring monocyte HLA-DR levels (< 8000 molecules/cell, indicating immune paralysis), lymphocyte subsets (CD4⁺ T cells < 200/μl, indicating CARS), and PD-L1 expression on peripheral blood monocytes (> 10% suggesting immune checkpoint overactivation). Organ injury is reflected by lactate levels (> 2 mmol/L, indicating a microcirculatory disturbance), creatinine levels (> 133 μmol/L, indicating AKI and necessitating a renal drug dose adjustment), and the PaO_2_/FiO_2_ ratio (< 300 mmHg, indicating ARDS and requiring prioritized control of pulmonary inflammation). For instance, in patients with viral pneumonia presenting with a cytokine storm, an IL-6 concentration > 300 pg/ml combined with a PaO_2_/FiO_2_ ratio < 200 mmHg supports the use of tocilizumab plus baricitinib [193]. If the concentration of IL-6 decreases to 50 pg/ml after 72 h but CD4⁺ T cell counts remain below 300/μl, tocilizumab should be discontinued and replaced with rIL-7 to mitigate the risk of secondary infections [107].

### Clinical challenges and solutions in balanced management

Despite the remarkable success in preclinical models, most anti-inflammatory therapies targeting cytokine storms (such as anti-TNF-α, anti-IL-1β, and anti-endotoxin agents) have failed to significantly reduce mortality in large Phase 3 sepsis clinical trials. We argue that this failure is not because the concept of a “cytokine storm” is incorrect, but may be explained by several key factors.

First, patient heterogeneity presents a major challenge. CRS is not a uniform disease but a clinically and immunologically heterogeneous syndrome. Emerging evidence supports the existence of distinct immune phenotypes in critically ill patients, particularly those with sepsis or ARDS [194–197]. A series of studies has systematically characterized these phenotypes. In a 2020 cluster analysis of more than 600 patients with ARDS, 2 major immunological subphenotypes were identified. A hyperinflammatory subtype was characterized by elevated levels of proinflammatory cytokines, increased use of vasopressors, and worse clinical outcomes. In addition, a hypoinflammatory subtype characterized by lower levels of inflammatory markers and a relatively better prognosis was identified [194]. These subphenotypes were further validated in other viral pneumonia cohorts, where the hyperinflammatory profile was associated with increased mortality and a greater likelihood of response to immunosuppressive therapies such as dexamethasone [195, 196]. Moreover, a comprehensive study extended this framework to sepsis, demonstrating that the early identification of immune subphenotypes using routinely available biomarkers (e.g., CRP levels, IL-6 levels, and lymphocyte count) could guide more precise immunomodulatory therapy. In this study, patients with hyperinflammatory sepsis benefited from anti-cytokine therapies, while those with hypoinflammatory or immunosuppressive profiles were more likely to develop secondary infections and potentially benefit from immune-stimulating agents [197]. Another representative example is the UK PANTHER trial (phase 2; Precision medicine adaptive network trial in hypoxemic acute respiratory failure). It uses a Bayesian adaptive platform design to evaluate novel interventions and to identify patient subgroups that may benefit from therapies such as simvastatin and baricitinib [198]. Compared with traditional fixed-design trials, adaptive platform trials are more flexible and efficient. They use interim data to modify randomization ratios, add or drop arms, and make other predefined adjustments, thereby accelerating learning and increasing the chance of detecting true treatment effects.

Second, the timing of the intervention is critical. The immune response to sepsis evolves dynamically, often progressing from an early hyperinflammatory phase to a state of immunosuppression dominated by a CARS within days. Regarding the treatment timing, a "dynamic monitoring and stepwise adjustment" strategy is recommended. For example, IL-6 and monocyte HLA-DR levels should be measured every 12 h in septic patients. If the concentration of IL-6 is > 100 pg/ml and the HLA-DR level is < 8000 molecules/cell, a combined regimen of a "low-dose anti-inflammatory agent and low-dose immune activator" (e.g., 100 mg/d hydrocortisone and 150 μg/d GM-CSF) should be adopted to avoid excessive intervention with a single drug [145, 189–192].

Third, a major reason for the failure of these therapies is that current animal models cannot fully replicate the dynamics and magnitude of the cytokine storm in humans. Future research should focus on improving animal models to achieve reliable clinical translation. Primary humanized mice, TLR4-mutant mice, or larger animals (sheep, nonhuman primates) that more closely resemble human physiological responses should be used to more accurately mimic human immune responses. Additionally, "secondary hit" models can be used to reproduce post-sepsis immunosuppression and secondary infections. Lastly, factors such as age, sex, comorbidities, and supportive therapies (vasopressors, mechanical ventilation) should be incorporated into the models to increase their similarity to clinical scenarios.

## Summary and outlook

The cytokine storm represents the "double-edged sword" nature of the immune response, where the mechanisms designed for host defense cause self-destructive systemic inflammation. Our description focused on the central mechanism whereby initial triggers (PAMPs, DAMPs, and inflammatory cell death) dysregulate the amplification cascade and drive the progression to multiple organ damage. While the currently employed therapies demonstrate efficacy, their efficacy is often limited by patient heterogeneity, timing challenges, and a lack of precision in treatment. The future of cytokine storm management lies in precision treatment and requires a paradigm shift toward immune phenotyping for patient stratification; the use of cutting-edge technologies (such as multiomics and artificial intelligence) to discover new biomarkers and therapeutic targets; and the development of next-generation targeted therapies targeting specific cytokines, cell death pathways, and immune cell functions. By focusing on these research hotspots, including stratified medicine, novel targets, advanced technologies, and combination/sequenced therapies, we can transform the cytokine storm from a frequently lethal complication to a manageable syndrome, ultimately prolonging survival and improving long-term recovery for patients with severe infections and sepsis. This improved treatment requires both basic immunology research and innovative clinical trials.

## Supplementary Information

**Additional file 1**. Search strategies. **Table 1** Basic characteristics for the ongoing RCTs.

## Data Availability

Not applicable.

## Abbreviations

ADM: Adrenomedullin
AKI: Acute kidney injury
ARDS: Acute respiratory distress syndrome
CAR-T: Chimeric antigen receptor T-cell
CARS: Compensatory anti-inflammatory response syndrome
COVID-19: Coronavirus Disease 2019
CRP: C-reactive protein
DAMPs: Damage-associated molecular patterns
DCs: Dendritic cells
DIC: Disseminated intravascular coagulation
GCs: Glucocorticoids
GM-CSF: Granulocyte-macrophage colony-stimulating factor
GVHD: Graft-versus-host disease
HLA-DR: Human leukocyte antigen-DR isotype
HLH: Hemophagocytic lymphohistiocytosis
IFN: Interferon
IL: Interleukin
JAK: Janus kinase
LPS: Lipopolysaccharide
MAPK: Mitogen-activated protein kinase
MAS: Macrophage activation syndrome
MDSCs: Myeloid-derived suppressor cells
MODS: Multiple organ dysfunction syndrome
NF-κB: Nuclear factor-kappa B
NLRs: NOD-like receptors
NLRP3: NOD-like receptor thermal protein domain containing protein 3
PAMPs: Pathogen-associated molecular patterns
PD-1: Programmed cell death protein-1
PRRs: Pattern recognition receptors
rIL-7: Recombinant interleukin-7
RCTs: Randomized controlled rials
RLRs: RIG-I-like receptors
SARS-CoV-2: Severe acute respiratory syndrome coronavirus 2
TGF-β: Transforming growth factor-β
Th: T helper type
TLRs: Toll-like receptors
TNF-α: Tumor necrosis factor-α
Tregs: Regulatory T cells
TREM-1: Triggering receptor expressed on myeloid cells-1
TTM: Targeted temperature management
