# Supplementary material for: The “cytokine storm” in infection and sepsis: win the battle but lose the war
Source: Mil Med Res. 2026 Jan 12;12:95. doi: 10.1186/s40779-025-00678-0 (PMC12794442; doi:10.1186/s40779-025-00678-0)
Supplement: Supplementary file 1 — Additional file 1. Search strategies. Table 1 Basic characteristics for the ongoing RCTs. [file 40779_2025_678_MOESM1_ESM.pdf]

Search strategies

#1. Cytokine storm

#2. Cytokine release syndrome

#3. Hypercytokinemia

#4. Systemic inflammatory response syndrome

#5. Macrophage activation syndrome

#6. Hemophagocytic lymphohistiocytosis

#7. Inflammatory storm

#8. Overactive immune response

#9. Hyperinflammation

#10. Uncontrolled inflammation

#11. Excessive immune response

#12. Inflammatory cascade

#13. Interleukin-1 inhibitors

#14. IL-1 Antagonists

#15. IL-1 inhibitors

#16. IL-1 inhibitor

#17. Anti-IL-1 agents

#18. IL-1 blocking agents

#19. IL-1 receptor antagonists

#20. Anakinra

#21. Kineret

#22. Canakinumab

#23. Ilaris

#24. Rilonacept

#25. Arcalyst

#26. Bermekimab

#27. Xilonix

#28. Gevokizumab

#29. IL-6 receptor inhibitors

#30. Anti-IL-6R antibodies

#31. IL-6 receptor blockers

#32. IL-6R monoclonal antibodies

#33. Anti-IL-6 receptor antibodies

#34. Interleukin-6 receptor antagonists

#35. IL-6R antagonists

#36. Tocilizumab

#37. Actemra

#38. RoActemra

#39. Sarilumab

#40. Kevzara

#41. JAK Inhibitors

#42. JAK antagonists

#43. JAK blockers

#44. Janus kinase inhibitors

#45. JAK-STAT inhibitors

#46. JAK-STAT pathway inhibitors

#47. JAK tyrosine kinase inhibitors

#48. Kinase inhibitors targeting JAKs

#49. JAK pathway inhibitors

#50. JAK1 inhibitors

#51. JAK2 inhibitors

#52. JAK3 inhibitors

#53. Pan-JAK inhibitors

#54. Tofacitinib

#55. Xeljanz

#56. Ruxolitinib

#57. Jakafi

#58. Jakavi

#59. Baricitinib

#60. Olumiant

#61. Upadacitinib

#62. Rinvoq

#63. Fedratinib

#64. Inrebic

#65. Peficitinib

#66. Smyraf

#67. Filgotinib

#68. Jyseleca

#69. Abrocitinib

#70. Cibinqo

#71. Deucravacitinib

#72. Sotyktu

#73. Caspase-1 Inhibitors

#74. Interleukin-1beta Converting Enzyme inhibitors

#75. Inhibitors of interleukin-1 converting enzyme

#76. IL-1β Converting Enzyme inhibitors

#77. Caspase-1 antagonists

#78. Caspase-1 blocking agents

#79. Inflammasome inhibitors

#80. Caspase-1 blockers

#81. Caspase-1 targeting agents

#82. Inhibitors of inflammatory caspase-1

#83. Caspase-1 catalytic inhibitors

#84. Belnacasan

#85. VX-765

#86. Pralnacasan

#87. VX-740

#88. Corticosteroids

#89. Steroids

#90. Cortisol

#91. Cortisone

#92. Hydrocortisone

#93. Prednisolone

#94. Prednisone

#95. Methylprednisolone

#96. Triamcinolone

#97. Dexamethasone

#98. Betamethasone

#99. Blood purification

#100. Blood cleansing

#101. Hemopurification

#102. Blood filtration

#103. Extracorporeal blood treatment

#104. Plasma exchange

#105. Seraph® 100

#106. CytoSorb

#107. oXiris

#108. Recombinant IL-7

#109. Recombinant human interleukin-7

#110. Recombinant interleukin-7

#111. Recombinant human IL-7

#112. IL-7 cytokine therapy

#113. rhIL-7

#114. rIL-7

#115. CYT107

#116. Efineptakin alfa

#117. PTX-100

#118. GM-CSF

#119. Granulocyte-Macrophage Colony-Stimulating Factor

#120. Colony-Stimulating Factor 2

#121. Granulocyte–macrophage CSF

#122. CSF-GM

#123. MGI-2

#124. Sargramostim

#125. CSF2

#126. Molgramostim

#127. IFN-γ

#128. Interferon-gamma

#129. Interferon gamma

#130. Interferon-γ

#131. Type II Interferon

#132. Actimmune

#133. Anti-PD-1

#134. PD-1 inhibitors

#135. Programmed cell death protein 1 inhibitors

#136. Anti-PD-1 antibodies

#137. PD-1 blocking antibodies

#138. PD-1 antagonists

#139. Immune checkpoint inhibitors targeting PD-1

#140. Anti-programmed cell death protein 1 antibodies

#141. PD-1 blockade

#142. Nivolumab

#143. Opdivo

#144. Pembrolizumab

#145. Keytruda

#146. Cemiplimab

#147. Libtayo

#148. Dostarlimab

#149. Jemperli

#150. Toripalimab

#151. Mesenchymal Stem Cells

#152. Mesenchymal Stromal Cells

#153. Mesenchymal Progenitor Cells

#154. Mesenchymal Stromal Progenitors

#155. Multipotent Mesenchymal Stromal Cells

#156. Mesenchymal Stem Cell-like Cells

#157. Alofisel

#158. Darvadstrocel

#159. Temcell

#160. Ryoncil

#161. Cx601

#162. MSC-NP

#163. ExoFlo

#164. MSC-EVs

#165. OR #1-#164

Table 1 Basic characteristics for the ongoing RCTs

Trial ID	Date of registration	Source of register	Sample size	Phase	Countries	Disease	Treatment
NCT06597396	20240912	ClinicalTrials.gov	90	Phase 2	United States	COVID-19	Abrocitinib
NCT05926505	20230628	ClinicalTrials.gov	182	Phase 2/Phase 3	Germany, Greece, Italy, Spain	COVID-19	Anakinra
NCT05914454	20230613	ClinicalTrials.gov	36	Phase 2	Italy	Acute respiratory distress syndrome	Anakinra
NCT05267821	20220224	ClinicalTrials.gov	500	Phase 2/Phase 3	United States	Sepsis	Anakinra
EUCTR2020-005828-11-IT	20210202	EUCTR	600	Phase 3	Greece, Italy	COVID-19	Anakinra
EUCTR2020-005828-11-GR	20201209	EUCTR	600	Phase 3	Greece	COVID-19	Anakinra
EUCTR2020-001825-29-ES	20200505	EUCTR	180	Phase 2/Phase 3	Spain	COVID-19	Anakinra
EUCTR2020-001636-95-GB	20200504	EUCTR	40	Phase 2	United Kingdom	COVID-19	Anakinra
EUCTR2020-001739-28-BE	20200410	EUCTR	210	Phase 2	Belgium	COVID-19	Anakinra
NCT04603742	20201021	ClinicalTrials.gov	170	Phase 2	United States	COVID-19	Anakinra
NCT04324021	20200325	ClinicalTrials.gov	16	Phase 2/Phase 3	United States, Italy	COVID-19	Anakinra, emapalumab
NCT06631287	20241004	ClinicalTrials.gov	550	Phase 3	United States	COVID-19	Baricitinib

Trial ID	Date of registration	Source of register	Sample size	Phase	Countries	Disease	Treatment
IRCT20231017059748N3	20231214	IRCT	40	Phase 3	Iran (Islamic Republic of)	Acute respiratory distress syndrome	Baricitinib
IRCT20220508054780N1	20220916	IRCT	15	Phase 3	Iran (Islamic Republic of)	COVID-19	Baricitinib
ISRCTN82395639	20230920	ISRCTN	2000	Phase 4	Scotland, United Kingdom	Critical illness	Baricitinib, imatinib
NCT05013034	20210818	ClinicalTrials.gov	300	Phase 2	United States	COVID-19	Basiliximab
NCT06692036	20241022	ClinicalTrials.gov	276	NA	China	Septic shock	Blood purification
NL-OMON55310	20200722	OMON	24	Phase 4	Netherlands	Bacterial infectious disorders, sepsis	Blood purification
NCT04997421	20210621	ClinicalTrials.gov	40	NA	Finland	Septic shock	Combined HA380 hemoadsorption and continuous veno-venous hemodiafiltration with Oxiris®-AN69 membranes
NCT04597034	20201003	ClinicalTrials.gov	35	NA	Mexico	COVID-19	Continuous renal replacement therapy with AN69-Oxiris membrane
ChiCTR2100053104	20211110	ChiCTR	80	NA	China	Sepsis	Continuous venovenous hemodiafiltration
ISRCTN49832318	20210902	ISRCTN	210	NA	Suriname	COVID-19	Convalescent plasma
NCT04195126	20180505	ClinicalTrials.gov	20	NA	Hungary	Major burn trauma	CytoSorb haemadsorption

Trial ID	Date of registration	Source of register	Sample size	Phase	Countries	Disease	Treatment
NCT06562803	20240815	ClinicalTrials.gov	60	NA	China	Hepatitis B-related acute-on-chronic liver failure and sepsis	Double plasma cytokine adsorption system with sequential low-dose plasma exchange
NCT05136183	20211108	ClinicalTrials.gov	200	NA	Thailand	Septic shock	HA-330 disposable hemoperfusion cartridge
NCT05182515	20211222	ClinicalTrials.gov	50	Phase 3	France	COVID-19	Plasma exchange
NCT05093075	20210719	ClinicalTrials.gov	80	Phase 2	Canada	Septic shock	Plasma exchange
IRCT20200416047099N2	20200704	IRCT	10	Phase 2	Iran (Islamic Republic of)	COVID-19	Plasma exchange
IRCT20220517054899N1	20220813	IRCT	40	Phase 3	Iran (Islamic Republic of)	COVID-19	Plasma exchange
IRCT20200317046797N5	20200419	IRCT	10	Phase 2	Iran (Islamic Republic of)	COVID-19	Hemodialysis
NCT04366908	20200425	ClinicalTrials.gov	517	Phase 2	Spain	Acute respiratory distress syndrome	Calcifediol
EUCTR2020-001370-30-GB	20200415	EUCTR	450	Phase 3	United States, France, Spain, Germany, Italy, United Kingdom	COVID-19	Canakinumab
IRCT20211017052786N1	20220118	IRCT	60	Phase 3	Iran (Islamic Republic of)	COVID-19	Colchicine
ACTRN12621001200875	20210908	ANZCTR	650	Phase 2	Australia	COVID-19	Dexamethasone

Trial ID	Date of registration	Source of register	Sample size	Phase	Countries	Disease	Treatment
EUCTR2021-001416-29-ES	20210526	EUCTR	200	Phase 3	Spain	COVID-19	Dexamethasone
IRCT20201015049030N1	20201107	IRCT	200	Phase 2	Iran (Islamic Republic of)	COVID-19	Dexamethasone
NCT04826822	20210328	ClinicalTrials.gov	440	Phase 3	Russian federation	COVID-19	Dexamethasone
NCT05491304	20220804	ClinicalTrials.gov	400	Phase 4	China	Pediatric hemophagocytic lymphohistiocytosis	Dexamethasone, etoposide, ruxolitinib
NCT05062681	20210924	ClinicalTrials.gov	60	Phase 2	Egypt	COVID-19	Dexamethasone, methylprednisolone
CTRI/2021/09/036099	20210901	CTRI	994	Phase 2	India	COVID-19	Doxazosin mesylate
RBR-9th6k46	20220815	ReBEC	NR	Phase 3	Brazil	Severe pneumonia	Hydrocortisone
NCT05354778	20220420	ClinicalTrials.gov	180	NA	Brazil	Severe pneumonia	Hydrocortisone
NCT04492228	20200725	ClinicalTrials.gov	100	NA	Italy	COVID-19	Eucaloric ketogenic nutrition
EUCTR2020-001750-22-GB	20200707	EUCTR	186	Phase 2	United Kingdom	COVID-19	Fostamatinib, ruxolitinib
IRCT20200502047268N1	20200516	IRCT	10	Phase 1/Phase 2	Iran (Islamic Republic of)	COVID-19	Granulocyte colony-stimulating factor
ISRCTN80791572	20231103	ISRCTN	3758	Phase 4	England, United Kingdom	Sepsis	Granulocyte-macrophage colony-stimulating factor
NCT04824222	20210326	ClinicalTrials.gov	366	Phase 3	Poland	COVID-19	Human fecal microbiota

Trial ID	Date of registration	Source of register	Sample size	Phase	Countries	Disease	Treatment
EUCTR2020-001558-23-IT	20200626	EUCTR	216	Phase 3	Italy	COVID-19	Hydroxychloroquine sulfate
NCT05986422	20230809	ClinicalTrials.gov	418	Phase 2	Germany	COVID-19	Methylprednisolone
IRCT20201229049872N1	20210422	IRCT	80	Phase 3	Iran (Islamic Republic of)	COVID-19	Methylprednisolone, colchicine
NCT05133635	20210111	ClinicalTrials.gov	NA	Phase 4	Turkey	COVID-19	Methylprednisolone, tocilizumab
NCT06496997	20240704	ClinicalTrials.gov	300	Phase 2/Phase 3	Egypt	Acute respiratory distress syndrome	Methylprednisolone, dexamethasone, hydrocortisone
NCT05768204	20221220	ClinicalTrials.gov	160	Phase 3	China	Severe pneumonia	Prednisolone acetate
NCT05444699	20211222	ClinicalTrials.gov	210	Phase 4	Finland	Bronchiolitis	Prednisolone sodium phosphate
EUCTR2020-005883-78-FR	20201223	EUCTR	220	Phase 3	France	COVID-19	Prednisolone, dexamethasone
NCT04979052	20201109	ClinicalTrials.gov	200	Phase 2	United States, Germany, Greece, Netherlands, Romania, Switzerland	Candidemia	Interferon gamma-1B
IRCT20200721048159N4	20210903	IRCT	100	Phase 3	Iran (Islamic Republic of)	COVID-19	Interferon-beta 1A, remdesivir
ISRCTN10449048	20231110	ISRCTN	282	Phase 2	England, Northern Ireland, United Kingdom	Critically illness	Interferon-gamma

Trial ID	Date of registration	Source of register	Sample size	Phase	Countries	Disease	Treatment
IRCT20180610040037N3	20230213	IRCT	120	Phase 3	Iran (Islamic Republic of)	Sepsis	Interferon-gamma
CTIS2022-502229-16-00	20221222	CTIS	132	Phase 3	France	Ventilator-acquired pneumonia	Interferon-gamma
IRCT20200317046797N3	20200411	IRCT	100	Phase 3	Iran (Islamic Republic of)	COVID-19	Intravenous immunoglobulin
NCT03651518	20180625	ClinicalTrials.gov	32	Phase 2	France	Inflammatory disease	Kineret, humira, stelara, cosentyx, roactemra, rituximab
ChiCTR2400088213	20240813	ChiCTR	80	Phase 1/Phase 2	China	Lung injury caused by major infectious diseases	Mesenchymal stem cells
NCT04444271	20200527	ClinicalTrials.gov	20	Phase 2	Pakistan	COVID-19	Mesenchymal stem cells
IRCT20200421047150N1	20200514	IRCT	90	Phase 2/Phase 3	Iran (Islamic Republic of)	COVID-19	Mesenchymal stem cells
EUCTR2020-001364-29-ES	20200427	EUCTR	26	Phase 1/ Phase 2	Spain	COVID-19	Mesenchymal stem cells
IRCT20080901001165N44	20200328	IRCT	12	Phase 1/ Phase 2	Iran (Islamic Republic of)	Acute respiratory distress syndrome	Mesenchymal stem cells
NCT04753476	20210211	ClinicalTrials.gov	48	Phase 2	Indonesia	COVID-19	Mesenchymal stem cells
EUCTR2020-002193-27-ES	20200714	EUCTR	20	Phase 2	Spain	COVID-19	Mesenchymal stromal cells

Trial ID	Date of registration	Source of register	Sample size	Phase	Countries	Disease	Treatment
NCT05354141	20220427	ClinicalTrials.gov	970	Phase 3	United States	Acute respiratory distress syndrome	Mesenchymal stem cell-derived extracellular vesicles
NCT05787288	20230327	ClinicalTrials.gov	240	Early Phase 1	China	COVID-19	Extracellular vesicles from mesenchymal stem cells
NCT05741099	20230210	ClinicalTrials.gov	20	Phase 1/Phase 2	China	COVID-19	Umbilical cord mesenchymal stem cells
NCT05682586	20230108	ClinicalTrials.gov	60	Phase 3	China	COVID-19	Umbilical cord mesenchymal stem cells
IRCT20211012052743N1	20211106	IRCT	60	Phase 3	Iran (Islamic Republic of)	COVID-19	Umbilical cord mesenchymal stem cells
CTRI/2021/09/036645	20210920	CTRI	50	Phase 1/ Phase 2	India	COVID-19	Umbilical cord mesenchymal stem cells
NCT04457609	20200527	ClinicalTrials.gov	40	Phase 1	Indonesia	COVID-19	Umbilical cord mesenchymal stem cells
NCT05969275	20230712	ClinicalTrials.gov	296	Phase 2	Canada	Septic shock	Umbilical cord-derived human mesenchymal stromal cells
IRCT20211012052735N1	20211105	IRCT	60	Phase 3	Iran (Islamic Republic of)	COVID-19	Methylene blue
NCT04483271	20200718	ClinicalTrials.gov	100	NA	Jordan	COVID-19	Omega3-FA
EUCTR2020-001437-12-ES	20200413	EUCTR	290	Phase 4	Spain	COVID-19	Pembrolizumab, tocilizumab, ciclosporin

Trial ID	Date of registration	Source of register	Sample size	Phase	Countries	Disease	Treatment
NCT04433546	20200528	ClinicalTrials.gov	54	Phase 2	United States	Acute respiratory distress syndrome	Pemziviptadil (PB1046)
IRCT20110425006280N11	20210127	IRCT	20	Phase 2	Iran (Islamic Republic of)	COVID-19	Pentoxifylline
IRCT20200721048159N3	20210603	IRCT	100	Phase 3	Iran (Islamic Republic of)	COVID-19	Pentoxifylline, colchicine
IRCT20220902055856N1	20220917	IRCT	42	Phase 3	Iran (Islamic Republic of)	Severe pneumonia	Propolis
NCT04280705	20200220	ClinicalTrials.gov	1062	Phase 3	United States, Denmark, Germany, Greece, Japan, Korea, Mexico, Singapore, Spain, United Kingdom	COVID-19	Remdesivir
ChiCTR2000039051	20201014	ChiCTR	60	Phase 0	China	Acute respiratory distress syndrome	Rhubarb enema, probiotic
NCT04348695	20200413	ClinicalTrials.gov	94	Phase 2	Spain	COVID-19	Ruxolitinib
CTRI/2020/09/027680	20200909	CTRI	402	Phase 3	Argentina, Brazil, Canada, Colombia, France, Germany, India, Italy, Mexico, Panama, Peru, Russian Federation, South Africa, Spain, Turkey, United Kingdom, United States	COVID-19	Ruxolitinib

Trial ID	Date of registration	Source of register	Sample size	Phase	Countries	Disease	Treatment
IRCT20210408050899N1	20210525	IRCT	15	Phase 3	Iran (Islamic Republic of)	COVID-19	Tocilizumab
ISRCTN46454974	20240118	ISRCTN	152	Phase 2	England, Scotland, United Kingdom	COVID-19	Tocilizumab
IRCT20151227025726N29	20220216	IRCT	50	Phase 2/Phase 3	Iran (Islamic Republic of)	COVID-19	Tocilizumab
EUCTR2020-001707-16-ES	20200722	EUCTR	60	Phase 2	Spain	COVID-19	Tocilizumab
EUCTR2020-001854-23-IT	20200626	EUCTR	1400	Phase 2/Phase 3	Italy	COVID-19	Tocilizumab
NCT04412772	20200601	ClinicalTrials.gov	300	Phase 3	United States	COVID-19	Tocilizumab
IRCT20200510047383N1	20200515	IRCT	100	Phase 3	Iran (Islamic Republic of)	COVID-19	Tocilizumab
EUCTR2020-001903-17-ES	20200515	EUCTR	120	Phase 3	Spain	COVID-19	Tocilizumab
EUCTR2020-001827-15-ES	20200505	EUCTR	72	Phase 3	Spain	COVID-19	Tocilizumab
NCT04377750	20200423	ClinicalTrials.gov	500	Phase 4	Israel	COVID-19	Tocilizumab
EUCTR2020-001767-86-IE	20200415	EUCTR	90	Phase 2	Ireland	COVID-19	Tocilizumab
IRCT20200406046968N1	20200414	IRCT	10	Phase 2/Phase 3	Iran (Islamic Republic of)	COVID-19	Tocilizumab

Trial ID	Date of registration	Source of register	Sample size	Phase	Countries	Disease	Treatment
CTRI/2021/05/033703	20210520	CTRI	188	Phase 3	India	COVID-19	Tocilizumab
EUCTR2020-001160-28-ES	20200413	EUCTR	24	Phase 2	Spain	COVID-19	Tocilizumab, pembrolizumab
IRCT20190804044429N7	20211027	IRCT	240	Phase 3	Iran (Islamic Republic of)	COVID-19	Tofacitinib
NCT04476745	20200716	ClinicalTrials.gov	100	NA	Jordan	COVID-19	Vitamin D3

ANZCTR Australian New Zealand Clinical Trials Registry, *COVID-19* Coronavirus Disease 2019*, CTIS* Clinical Trials Information System, *CTRI* Clinical Trials Registry-India, *EUCTR* European Union Clinical Trials Register, *IRCT* lranian Registry of Clinical Trials*, ISRCTN* International Standard Randomised Controlled Trial Number, *NA* not applicable, *NR* not report, *OMON* Overview of Medical Research in the Netherlands, *ReBEC* Registro Brasileiro de Ensaios Clinicos
